# The Mucolipin TRPML2 Channel Enhances the Sensitivity of Multiple Myeloma Cell Lines to Ibrutinib and/or Bortezomib Treatment

**DOI:** 10.3390/biom12010107

**Published:** 2022-01-09

**Authors:** Giorgio Santoni, Consuelo Amantini, Federica Maggi, Oliviero Marinelli, Matteo Santoni, Maria Beatrice Morelli

**Affiliations:** 1 School of Pharmacy, University of Camerino, 62032 Camerino, Italy; oliviero.marinelli@unicam.it; 2 School of Biosciences and Veterinary Medicine, University of Camerino, 62032 Camerino, Italy; consuelo.amantini@unicam.it (C.A.); federica.maggi@uniroma1.it (F.M.); 3 Department of Molecular Medicine, Sapienza University, 00185 Rome, Italy; 4 Medical Oncology Unit, Hospital of Macerata, 62100 Macerata, Italy; mattymo@alice.it

**Keywords:** multiple myeloma, TRPML2, Ibrutinib, Ibrutinib resistance, Bortezomib

## Abstract

Multiple myeloma (MM) is a haematological B cell malignancy characterised by clonal proliferation of plasma cells and their accumulation in the bone marrow. The aim of the present study is the evaluation of biological effects of Ibrutinib in human MM cell lines alone or in combination with different doses of Bortezomib. In addition, the relationship between the expression of TRPML2 channels and chemosensitivity of different MM cell lines to Ibrutinib administered alone or in combination with Bortezomib has been evaluated. By RT-PCR and Western blot analysis, we found that the Ibrutinib-resistant U266 cells showed lower TRPML2 expression, whereas higher TRPML2 mRNA and protein levels were evidenced in RPMI cells. Moreover, TRPML2 gene silencing in RPMI cells markedly reverted the effects induced by Ibrutinib alone or in combination with Bortezomib suggesting that the sensitivity to Ibrutinib is TRPML2 mediated. In conclusion, this study suggests that the expression of TRPML2 in MM cells increases the sensitivity to Ibrutinib treatment, suggesting for a potential stratification of Ibrutinib sensitivity of MM patients on the basis of the TRPML2 expression. Furthermore, studies in vitro and in vivo should still be necessary to completely address the molecular mechanisms and the potential role of TRPML2 channels in therapy and prognosis of MM patients.

## 1. Introduction

Multiple myeloma (MM), classified as a plasma cell malignancy, represents approximately 10% of all haematological malignancies. At the time of diagnosis, the average age of people with MM is 66–70 years [1]. Population studies demonstrated that age is strongly associated with MM progression and regimen strategy [2,3]. Survival is higher in young people, both for the possibility of carrying out autologous stem cell transplantation (ASCT) and for the better tolerability of the drug during adjuvant therapy to ASCT or non-transplanted systemic therapy. MM evolves from a pre-malignant stage monoclonal gammopathy of undetermined significance (MGUS) [4,5] and an asymptomatic intermediate stage called smoldering multiple myeloma (SMM) [6,7]. Following clonal evolution and the development of chemoresistance, MM can evolve into an aggressive disease known as plasma cell leukemia (PCL), independent of the bone marrow, in which MM cells proliferate, causing an increase in plasma cells (≥20%) and often plasmacytomas [8]. In addition, MM progression also leads to bone destruction, hypercalcemia and renal insufficiency, and may result in patient lethality.

Although MM is still incurable, quality of life and survival of patients have been now improved significantly thanks to new therapies. Treatment of MM has progressed in the last decade, with the introduction of proteasome inhibitors (Bortezomib and Carfilzomib) and immunomodulatory drugs (Lenalidomide and Thalidomide) [9,10]. Inhibition of the proteasome promotes death by apoptosis because it causes the accumulation of misfolded and ubiquitinated proteins and does not allow the degradation of pro-apoptotic factors [11].

Bortezomib, a potent inhibitor of proteasome, was approved for MM treatment by the FDA in 2003. Treatment is associated with clinical beneficial effects on myeloma bone disease, by inhibiting osteoclastogenesis and stimulating the osteoblastogenesis [12]. However, the development of Bortezomib-resistance is unfortunately unavoidable and the disease, at present, remains incurable [13]. Thus, MM patients inevitably relapse on initial treatment regimens, and novel combination therapies are needed. 

Ibrutinib (previously known as PCI-32765) is a first-in-class, once-daily inhibitor of Bruton’s tyrosine kinase (BTK), an enzyme involved in growth and survival of MM cells. It has shown clinical activity in chronic lymphocytic leukaemia and Waldenstrom’s macroglobulinemia, as well as in MM [14,15]. The BTK, expressed at high levels in CD19^+^B cells, CD14^+^ monocytes and B-lymphoblasts, has a key role in B cell development and plasma cell (PC) differentiation. Ibrutinib, via BTK inhibition, interferes with the intracellular B cell signaling as well as limits the survival of malignant B cells by promoting apoptosis [16]. The B cell receptor (BCR) signaling pathway is a therapeutic target for the BTK inhibitor, Ibrutinib.

Ibrutinib is cytotoxic to malignant PCs from MM patients and treatment enhances the cytotoxic activity of Bortezomib and Lenalidomide. The Ibrutinib cytotoxicity in MM occurs via inhibition of NF-κB pathway; in fact, Ibrutinib stops the Ser536 phosphorylation of the p65 subunit of NF-κB, blocking its nuclear translocation, thus resulting in down-regulation of anti-apoptotic proteins such as Bcl-xL, FLIP(L) and survivin and activating caspase-mediated apoptosis in malignant PCs [17].

The BTK is an important factor for normal B cell differentiation and maturation and BTK-signalling cascade activates several downstream pathways upon antigen engagement by the BCR or TLRs activation. Within the BTK signalling pathway, activation of protein kinase C triggers the NF-kB of activated B cells to induce gene transcription [18].

Interestingly, the *MCOLN2* gene, which codifies for the mucolipin TRPML2 channel belonging to the transient receptor potential (TRP) ion channels, is among the genes targeted by the activation of the BTK pathway [19]. The TRPML2 mRNA is detectable by RT-PCR in lymph nodes, tonsils and spleen [20] and is also detected in A20 mature B lymphocytes, in several B lymphomas and in the 5T33 myeloma cell lines [19,21]. The TRPML2 is expressed in pre-B cell, mature B cell and plasma cell stages and it is also expressed in the T1 B-lymphocytes population expressing a functional pre-BCR or BCR, suggesting that it plays a specific role in B cell development [22]. In addition, the B cell lineage specific activator protein (BSAP), also known as paired box 5 (PAX5), required for normal B development [23], is the transcription factor regulating the *MCOLN2* gene expression [24]. The *MCOLN2* gene has been also found to be hypermethylated in the 5′ regulatory region and down-regulated in acute lymphoblastic B cell leukaemia [25]. 

Furthermore, Lindvall and colleagues showed that TRPML2 mRNA expression significantly increased in wild-type whole primary splenic B cells stimulated with anti-IgM or phorbol ester (PMA) plus ionomycin. Moreover, a four-fold decrease in the TRPML2 mRNA levels was evidenced in BTK-defective B cells, compared to wild-type splenic B cells. Thus, destruction of the BTK pathway significantly affected the *MCOLN2* gene expression [19].

The ion channels belonging to the TRP family are heterogeneously expressed in different cancer types [26,27]. Although TRP channels have been described in different hematological malignancy, information in MM is limited. Members of the TRP channel family control cancer cell fate by regulating apoptotic cell death and drug-resistance [28]. Malignant transformation of cells, resulting from aberrant differentiation, is often associated with alterations in TRP channel expression and therefore by atypical drug responses. In this regard, we have previously described that activation of TRPV2 channel, belonging to the vanilloid TRP subfamily, by Cannabidiol (CBD), a TRPV2 agonist, abrogates BCNU resistance in glioma stem-like cells [29,30]. Moreover, we have previously reported in MM patients, that TRPV2 is heterogeneously expressed in CD138^+^ plasma cells and CBD administration, alone or in synergy with Bortezomib, in TRPV2 expressing cells strongly inhibits growth, arrests cell cycle progression and induces the death of MM cells [31]. 

Thus, the aim of this work is to evaluate the effects of the BTK inhibitor, Ibrutinib, administered alone or in combination with Bortezomib in U266 and RPMI MM cell lines. Moreover, the modulatory effect of the TRPML2 expression on the sensitivity of MM cells to Ibrutinib and/or Bortezomib treatments will be investigated.

## 2. Materials and Methods

### 2.1. Cell Lines

RPMI8226 (RPMI) and U266 MM cell lines were from ATCC (LGC Standards, Milan, Italy). Cell lines were cultured in RPMI medium (Lonza, Milan, Italy) supplemented with 10% foetal bovine serum (FBS), 2 mM L-glutamine, 100 IU/mL penicillin, 100 μg streptomycin and 1 mM sodium pyruvate. All cell lines were maintained at 37 °C with 5% CO_2_ and 95% of humidity.

### 2.2. MTT Assay

A quantity of 1 × 10^4^/mL U266 cells and 6 × 10^3^/mL RPMI cells were plated in 96-well plates and treated with different doses of Ibrutinib (5, 12.5 and 25 µM) for 24 and 48 h alone and in combination with Bortezomib (1, 3, 6 ng/mL). At the end of the treatment, 0.8 mg/mL of MTT was added to the samples and incubated for 3 h. Medium was removed from the wells, the formazan crystals were dissolved with 100 µL/well of DMSO and the absorbance was read by microtiter plate spectrophotometer (BioTek Instruments, Winooski, VT, USA). Four replicates were used for each treatment. IC50 values, the drug concentration that induces 50% of cell growth inhibition, were calculated using GraphPad Prism^®^ 5.0a (GraphPad Software, San Diego, CA, USA). 

### 2.3. Cell Cycle Analysis

MM cell lines were incubated with Ibrutinib at concentration of 12.5 and 25 µM for 24 and 48 h. Cells were fixed in ice-cold 70% ethanol, treated for 30 min at 37 °C with 100 μg/mL ribonuclease A solution, stained for 30 min at room temperature with PI 20 μg/mL, and analysed by flow cytometry using linear amplification.

### 2.4. Annexin-V/PI Assay

To assess cell death, Annexin V and PI staining was used. Briefly, untreated cell and cells treated with Ibrutinib at different doses (12.5 and 25 µM), were incubated with 5 μL Annexin V-FITC or 20 μg/mL PI for 10 min at room temperature. The cells were then analysed by flow cytometry using CellQuest software.

### 2.5. Gene Expression Analysis

Total RNA extraction was obtained with the RNeasy Mini Kit (Qiagen), and cDNA was synthesized using the High-Capacity cDNA Archive Kit (Applied Biosystems, Foster City, PA, USA) according to the instructions. Quantitative RT-PCR was performed by using the IQ5 Multicolor real-time PCR detection system (BioRad, Hercules, CA, USA) and the RT2 SYBR^®^ Green qPCR Mastermix (QIAGEN). Human TRPML2 primers (forward 5′-34CGGCAGCCTTATCGTTTTCC-3′; reverse 5′-GCCATTGCATTTCTGACGGT-3′). GAPDH (used as housekeeping gene) primers (forward 5′-AGAAAAACCTGCCAAATATGATGAC-3′; reverse 5′-TGGGTGTCGCTGTTGAAGTC-3′;) were designed by Primer Express Software (PE Applied Biosystems) and purchased from Sigma Genosys (The Woodlands, TX, USA). The PCR parameters were 10 min at 95 °C followed by 40 cycles of 95 °C for 15 s and 60 °C for 40 s. All samples were assayed in triplicate. The 2^−^^ΔΔCt^ method was applied to calculate the relative gene expression level. 

### 2.6. Mitochondrial Transmembrane Potential (Δψ_m_) Assay

Δψ_m_ was evaluated by 5,50,6,60-tetrachloro-1,10,3,30-tetraehylbenzimidazolylcarbocyanineiodide (JC-1) staining. RPMI and U266 cells, treated with Ibrutinib 12.5 and 25 µM for 24 and 48 h, were incubated for 10 min at room temperature with 10 μg/mL of JC-1. Green (530 nm) and red (570 nm) emission fluorescences were recorded simultaneously. Samples were analysed using a FACScan cytofluorimeter with CellQuest software.

### 2.7. TRPML2 Gene Silencing

TRPML2 (siTRPML2) and siCONTROL non-targeting siRNA (siGLO, used as negative control) FlexiTube siRNA were from Qiagen (Milan, Italy). For gene silencing experiments, MM cell lines were plated at the density of 1.2 × 10^5^/mL and siTRPML2 or siGLO (150 ng) was added to the wells, following the HiPerfect transfection reagent protocol (Qiagen, Milan, Italy). No differences were observed comparing siGLO transfected with untransfected cells. 

### 2.8. Western Blot

Total lysates from RPMI and U266 cell lines were lysed in a lysis-buffer containing protease inhibitor cocktail (Sigma-Aldrich, Milan, Italy). Proteins were separated on 8% SDS polyacrylamide gel, transferred onto Hybond-C extra membranes (GE Healthcare, Milan, Italy) and blotted with the specific Abs. 5% low-fat dry milk, 5% bovine serum albumin (BSA) in phosphate-buffered saline 0.1% Tween 20 O/N were used to block non-specific binding sites. Membranes were incubated with the mouse anti-human TRPML2 (Santa Cruz Biotechnology, Dallas, TX, USA) or mouse anti-human GAPDH (Santa Cruz Biotechnology, Dallas, TX, USA) primary Abs for 1h at room temperature followed by HRP-conjugated anti-mouse Ab for 1h at room temperature. The detection was performed using the LiteAblot PLUS (EuroClone, Milan, Italy) kits, and densitometric analysis was carried out by a Chemidoc using the Quantity One software (Bio-Rad, Hercules, CA, USA). For quantification, GAPDH was used as loading control. One representative out of three independent experiments is shown. 

### 2.9. Statistical Analysis

The statistical significance was determined by Student’s *t*-test and by one way ANOVA. The statistical analysis of IC50 levels was performed using Prism 8.0 (GraphPad Software, San Diego, CA, USA). 

## 3. Results

### 3.1. Cytotoxic Effect of Ibrutinib on MM Cell Viability

**Figure 1:**
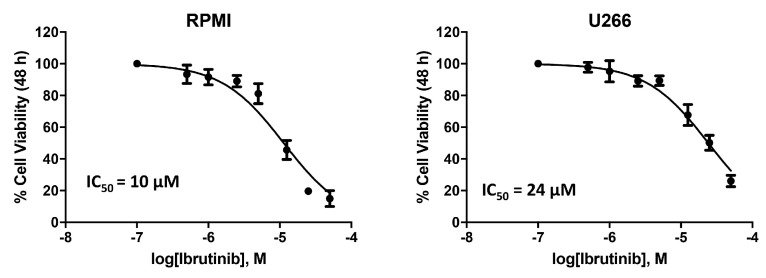
Ibrutinib treatment reduces cell viability in RPMI and U266 cell lines. Cell viability was evaluated by MTT in MM cells treated with Ibrutinib at different doses for 48 h.

We analysed the effects of the BTK inhibitor, Ibrutinib, on MM cell lines. The cytotoxic effect was evaluated by treating U266 and RPMI MM cells, with different doses of the drug. Cell viability was determined by MTT assay after 48 h of treatments (Figure 1). The RPMI cells with an IC_50_ of 10 μM demonstrated a higher sensitivity to Ibrutinib compared to U266 cells with an IC_50_ of 24 μM. Therefore, given the results obtained, we choose the dose of 12.5 and 25 µM of Ibrutinib for the subsequent experiments.

### 3.2. Ibrutinib Effects on Cell Cycle 

**Figure 2:**
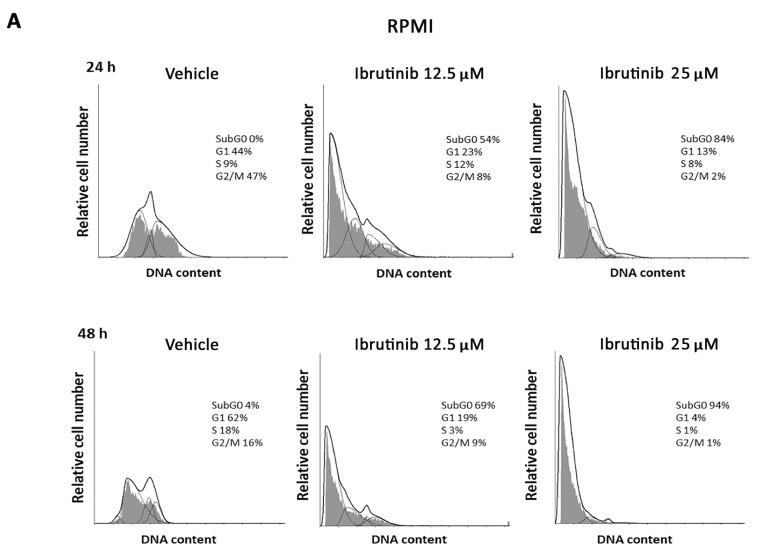

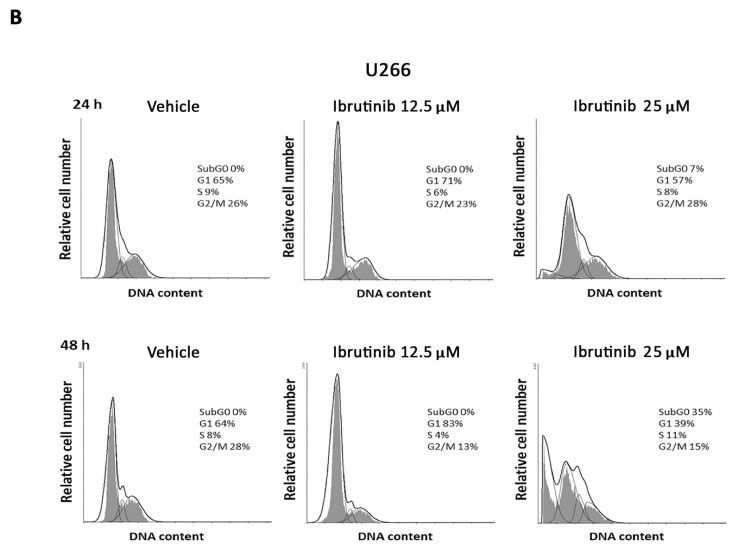
Representative cell cycle distribution in MM cells. (**A**) RPMI and (**B**) U266 cell lines were treated for 24 and 48 h with Ibrutinib. Data are one out of three separate experiments. Data are expressed as percentage of cells in each cell cycle phase.

The effect of different doses of Ibrutinib on cell cycle progression was analysed in RPMI and U266 cells. Results showed that in RPMI cells BTK inhibitor arrests the cell cycle at G1 phase and accumulates cells at sub-G0 phase, just at 12.5 µM after 24 h with 94% of cells in sub-G0 phase observed at 25 µM after 48 h (Figure 2A). U266 cells were more resistant to the Ibrutinib effects. In this cell line, no changes were observed after 24 h at any Ibrutinib doses, and the maximal effect (35% of cells in sub-G0 phase) was detected at 25 µM dose after 48 h (Figure 2B). Overall, these data suggest that Ibrutinib induces DNA fragmentation and G1 arrest in both MM cells, with RPMI more sensitive and U266 cells more resistant to its effects.

### 3.3. Ibrutinib Induces Apoptotic Cell Death in MM Cell Lines 

**Figure 3:**
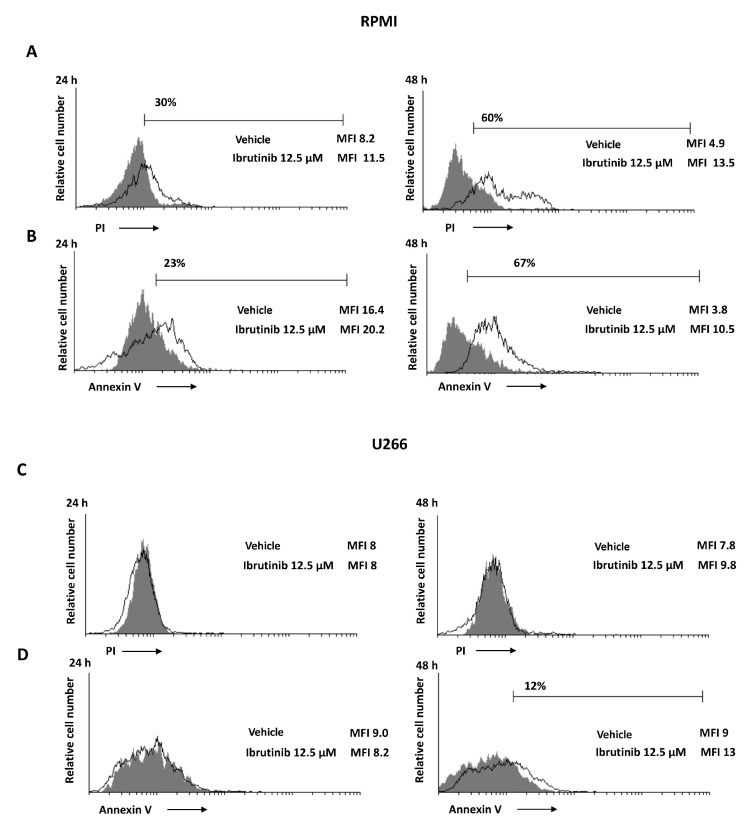
Ibrutinib induces cell death in MM cells. (**A**,**C**) PI incorporation was analysed by flow cytometry in RPMI and U266 treated with Ibrutinib for 24 and 48 h. Histograms are representative of one of three separate experiments. (**B**,**D**) Flow cytometric analysis was performed by Annexin V-FITC staining in MM cells treated as above described. Histograms are representative of one of three separate experiments. MFI = mean fluorescence intensity.

The capability of Ibrutinib at 12.5 µM to induce apoptotic cell death was evaluated. The results showed that Ibrutinib induces a PI positive signal in RPMI cell lines compared to vehicle-treated cells (Figure 3A); instead, no PI positive staining was evidenced in U266 cell lines (Figure 3C). Moreover, in order to evaluate a potential apoptotic cell death effect induced by Ibrutinib treatment, Annexin V staining was performed at the same condition tested for the PI assay. Annexin V positive signal was evident in RPMI cells (about 67%) (Figure 3B) and in U266 cell line (about 12%) (Figure 3D), suggesting that, although at different extension, both MM cell lines undergo apoptosis.

### 3.4. Ibrutinib Induces Mitochondrial Depolarization, Mainly in RPMI Cells 

**Figure 4:**
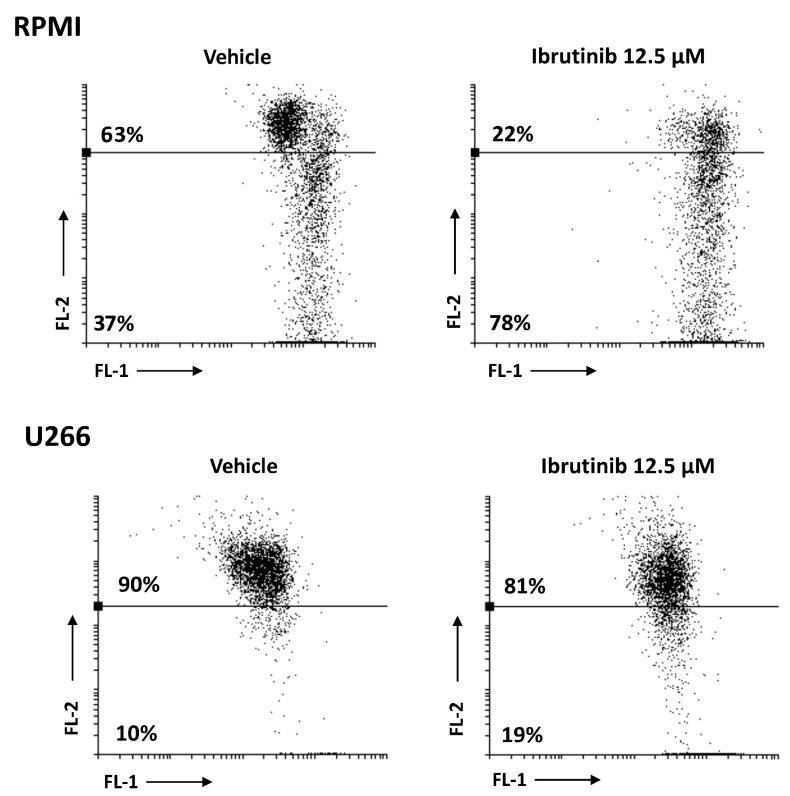
RPMI and U266 were treated with Ibrutinib and the Δψ_m_ changes were evaluated by JC-1 staining and biparametric FL-1/FL-2 flow cytometric analysis. Data are representative of one out of three separate experiments.

The effect of Ibrutinib on mitochondrial transmembrane potential (Δψ_m_) was detected using JC-1 staining. After 48 h exposure to 12.5 µM Ibrutinib, RPMI cells showed a marked drop of Δψ_m_ compared to vehicle-treated cells (about 78 vs. 37%), whereas a lower effect compared to vehicle treated cells (about 19 vs. 10%) was evident in U266 (Figure 4). No changes in Δψ_m_ were evident in vehicle-treated cells compared to untreated cells (data not shown).

### 3.5. Ibrutinib and Bortezomib in Combination Increase the Cytotoxicity in RPMI But Not in U266 Cells 

**Figure 5:**
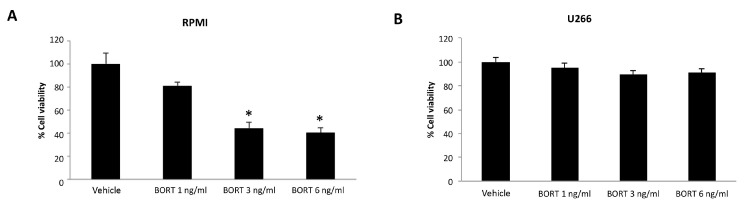
Cell viability assay in RPMI (**A**) and U266 (**B**) treated for 48 h with Bortezomib (BORT). Data are the mean ± SD of three different experiments. * *p* < 0.05 vs. vehicle-treated cells.

We first evaluated the effect of Bortezomib treatment for 48 h in both MM cell lines. We found that Bortezomib markedly inhibits the cell viability of RPMI cells [31], whereas no cytotoxic effect was observed at any dose in U266 cells (Figure 5A,B). 

**Figure 6:**
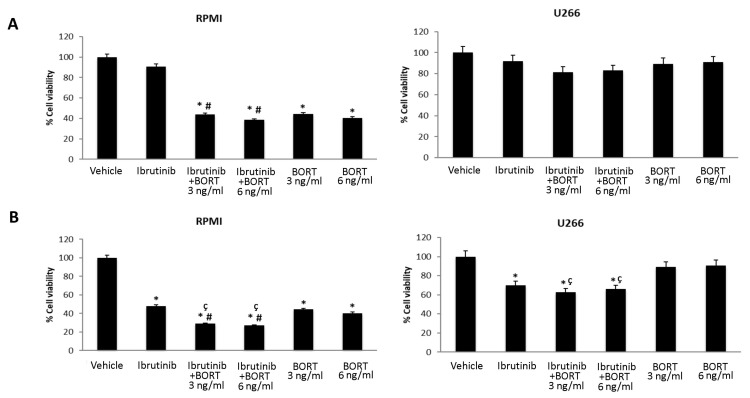
Cell viability assay in RPMI and U266 treated for 48 h with Bortezomib (BORT) in combination with Ibrutinib 5 µM (**A**) and 12.5 µM (**B**). Data are the mean ± SD of three different experiments. * *p* < 0.05 vs. vehicle, ^#^ *p* < 0.05 vs. Ibrutinib 12.5 µM, ^ç^ *p* < 0.05 vs. BORT.

Furthermore, we evaluated the effects of Bortezomib-Ibrutinib combination in both MM cell lines. U266 and RPMI cells were treated with Bortezomib 3 and 6 ng/mL and Ibrutinib 5 or 12.5 µM suboptimal doses alone and in combination for 48 h (Figure 6A,B). Ibrutinib at 5 µM in combination with Bortezomib at 3 ng/mL strongly increased the cytotoxic effect in RPMI cells, compared to Ibrutinib monotherapy (from 92% vs. 39%). More cytotoxic effects were observed at 12.5/3 or 12.5/6 Ibrutinib/Bortezomib ratio: from 48% to 30% or 25% vs. Ibrutinib and from 42% or 39% to 30% or 25% vs. Bortezomib 3 or 6 ng/mL, respectively. No major effects were observed at any Ibrutinib/Bortezomib combination in U266 cells (Figure 6A,B).

### 3.6. Expression of TRPML2 Channel in RPMI and U266 Cell Lines

**Figure 7:**
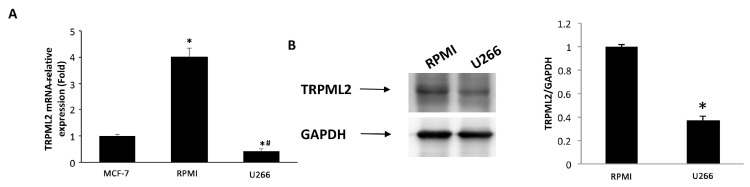
TRPML2 expression in MM cell lines. (**A**) The relative TRPML2 mRNA expression in RPMI and U266 cells was evaluated by qRT-PCR. MCF-7 breast cancer cell line has been used as positive control [32]. TRPML2 mRNA levels were normalized for glyceraldehyde-3-phosphate dehydrogenase (GAPDH) expression. Data are expressed as the mean ± SD. * *p* < 0.01 vs. MCF-7, ^#^ *p* < 0.01 vs. RPMI. (**B**) Total lysates were separated on 8% SDS-PAGE and probed with anti-TRPML2 Ab. Blots are representative of one of three separate experiments. Densitometry values were normalized to GAPDH, which was used as loading control. Data are expressed as the mean ± SE of three separate experiments. * *p* < 0.05.

Then, the expression of TRPML2 mRNA in RPMI and U266 cell lines was evaluated by qRT-PCR using the TaqMan^®^ Array Plates. High TRPML2 mRNA expression was evidenced in RPMI cells, whereas lower TRPML2 mRNA expression was observed in U266 cells (Figure 7A). These data were confirmed by Western blot analysis using a mouse anti-human TRPML2 antibody. A band of 68 kDa molecular weight, likely corresponding to TRPML2 proteins, was found. A lower TRPML2 protein expression was present in U266 compared with RPMI cells (Figure 7B).

### 3.7. TRPML2 Silencing in RPMI Cells Reduces the Cytotoxic Effect of Ibrunitinb Alone or in Combination with Bortezomib 

**Figure 8:**
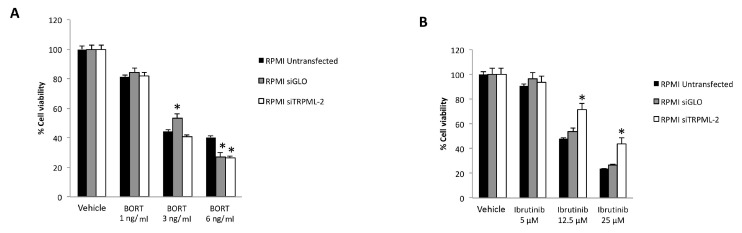
Cell viability assay in untransfected, siGLO and siTRPML2 RPMI cells treated for 48 h with Bortezomib (BORT) (**A**) or Ibrutinib (**B**). Data are the mean ± SD of three different experiments. * *p* < 0.05 vs. untreated cells.

**Figure 9:**
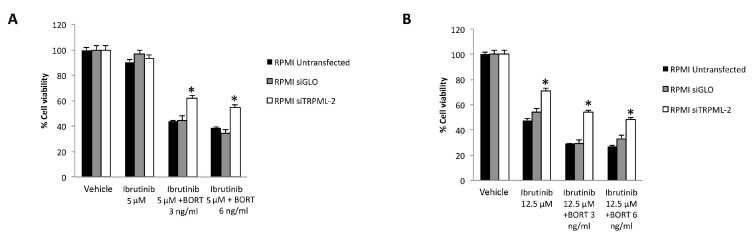
Cell viability assay in untransfected, siGLO and siTRPML2 RPMI cells treated for 48 h with Ibrutinib 5 µM (**A**) or 12.5 µM (**B**) in combination with Bortezomib (BORT). Data are the mean ± SD of three different experiments. * *p* < 0.05 vs. siGLO and untreated cells.

In order to investigate the role of TRPML2 on Ibrutinib-induced cytotoxic effects in higher TRPML2 expressing RPMI cells, we silenced the TRPML2 gene by RNA interference. RPMI cells were treated with the specific siRNA targeting the TRPML2 (siTRPML2) or siCONTROL non-targeting siRNA (siGLO) for 48 h. The silencing was evaluated by RT-PCR and Western blot analysis. After 48 h of transfection, a marked reduction in both TRPML2 mRNA and protein levels was evidenced in siTRPML2 compared to siGLO RPMI cells; no major differences were observed between siGLO and untransfected RPMI cells (Supplementary Figure S1). Then, the effect of TRPML2 silencing on the cytotoxic effect induced by Ibrutinib and Bortezomib, alone or in combination was evaluated at 48 h by MTT assay (Figure 8 and Figure 9). No major differences were found in Bortezomib-treated siTRPML2 compared to siGLO-treated RPMI cells (Figure 8A). On the other hand, TRPML2 silencing in RPMI cells partially reverted the Ibrutinib-induced cytotoxic effect at 12.5 and 25 µM dose (Figure 8B). 

Finally, the silencing of TRPML2 in RPMI cells partially reverted the cytotoxic effect induced by Ibrutinib at 5 or 12.5 µM suboptimal dose in combination with Bortezomib at 3 or 6 ng/mL (Figure 9A,B).

Overall, these results suggest that Ibrutinib-induced cytotoxicity is partially TRPML2-dependent.

## 4. Discussion

MM, the second most common blood cancer, is characterized by plasma cells clonal proliferation in the bone-marrow microenvironment, secretion of monoclonal proteins in blood or urine, anaemia, bone lesions, hypercalcemia and renal lesions [33]. In MM therapy, the use of the proteasome inhibitors, such as Bortezomib and Carfilzomib, and/or immunomodulators, such as Lenalidomide and Pomalidomide [15] have increased the survival of MM patients; however, despite these therapies, the MM remains an incurable disease [4,15]. Stratification of MM patients for different susceptibility to the therapeutic protocols is further needed to improve the clinical efficiency of chemotherapy in MM patients. 

At present, preclinical data suggest supra-additive or synergic effects between Ibrutinib and proteasome inhibitors against MM. In trial of phase 1, the combination of Ibrutinib with Carfilzomib/Dexamethasone (CA/DE) in patients with relapsed or relapsed/refractory multiple myeloma (RRMM) have been used [34]. Overall response rate (ORR) was 67% (very good partial response, 21%; stringent complete response, 2%) with an additional 9% minimal response. Median progression-free survival (PFS) was 7.2 months; however, the median overall survival (OS) was not reached. A phase 2 study of Ibrutinib in combination with Bortezomib and Dexamethasone in RRMM patients (NCT02902965) [35], showed that the median follow-up was 19.6 months, PFS: 8.5 months (95% CI: 6.2–10.8); OS was not reached. The ORR was 57% (95% CI: 45–68), and the median duration of response was 9.5 months (95% CI: 6.9–10.6). Finally, in another study of phase 1/2b with Ibrutinib combined with CA/DE in RRMM patients [36], the ORR was 71% (stringent complete response (CR) and CR: 3% each) in the RP2D population. The median duration of clinical benefit and median duration of response were both 6.5 months, PFS was 7.4 months, and OS was 35.9 months. Ibrutinib plus Carfilzomib showed anti-tumour action within the predictable efficacy range and the combination was well tolerated.

Herein, we found that Ibrutinib is more effective in RPMI cell line compared to U266 cell line. Similarly, Bortezomib inhibits the RPMI MM cell viability already at 1 ng/mL dose, whereas no major effect was observed in U266 cell line.

Thus, Ibrutinib administration at 12.5 and 25 µM dose for 24 h reduces the RPMI cells in G1 phase with a marked subG0 cells accumulation, which increase at 48 h after treatment. On the contrary U266 cells were arrested in G1 phase at low Ibrutinib dose (12.5 μM), whereas a subG0 accumulation was observed at higher dose after long exposition time. Finally, an increased dose-, time- and mitochondrial-dependent Annexin-V-positive apoptotic cells were evidenced in RPMI cells, whereas a low number of Annexin-V positive cells with minimal mitochondrial depolarization was evidenced in Ibrutinib-treated U266 cells. 

Finally, Ibrutinib at 5 and 12.5 μM, used in combination with Bortezomib at 3 and 6 ng/mL for 48 h, increased the cytotoxic activity in RPMI cells, compared to Ibrutinib or Bortezomib administered alone. By contrast, no major effect was observed in U266 cells at any Ibrutinib/Bortezomib drug combination.

Overall, our results demonstrated that Ibrutinib alone or in combination with Bortezomib, differently affected the viability, cell cycle and mitochondrial-dependent apoptotic cell death in RPMI compared to U266 MM cells, with RPMI more sensitive and U266 more resistant to Ibrutinib-induced cytotoxic effects.

Ibrutinib is a specific BTK inhibitor in B-lymphocytes; however, BTK has not only been implicated in B cell malignancies, but surprisingly it has recently been connected with GBM tumorigenesis. Colony formation and migration were markedly reduced in GBM cell line when BTK is down-regulated. BTK-silenced cells display a decline in the Akt/mTOR signalling [37]. Ibrutinib inhibits the proliferation, migration/invasion in glioma cells. It is required for EGFR-induced NF-κB activation and in different pathways of drug resistance [38,39]. Overexpression of active Akt decreases Ibrutinib-induced autophagy, while inhibition of Akt by LY294002 treatment, enhances the Ibrutinib-induced autophagy [40]. High expression of BTK is a prognostic marker for poor survival in patients with glioma. In regard to GBM, we have previously found that TRPML2 is expressed in glioma tissues and cell lines. TRPML2 levels increased with pathological grade; moreover, a role for TRPML2 in survival and proliferation of glioma cell lines has been reported [32]. Knock-down of TRPML2 in GBM cells abrogates Akt/mTOR and ERK signalling. High TRPML2 expression seems to have a pro-tumorigenic role in glioma progression [32].

Given the effects of Ibrutinib and TRPML2 in GMB, we hypothesized a role for TRPML2 also in MM. Indeed, we found that the high TRPML2 expression in RPMI is associated with the sensitivity to Ibrutinib-treatment; on the contrary the Ibrutinib resistance is associated with a reduced TRPML2 expression in U266 cells.

Finally, we silenced the TRPML2 gene in high TRPML2-expressing RPMI cells and the effect of Ibrutinib alone or in combination with Bortezomib was evaluated. No effects were observed in the Bortezomib-treated siTRPML2 vs. siGLO RPMI cells, suggesting the lack of any correlation between the Bortezomib effects and TRPML2 expression in RPMI cell line. On the other hand, the silencing of TRPML2 in RPMI cells partially reverts the cytotoxic effect induced by Ibrutinib alone or in combination with Bortezomib at different drug combinations, compared to siGLO RPMI cells. 

Overall, our in vitro data demonstrated that TRPML2 mediates Ibrutinib effects in MM cells, but further studies will be necessary to complete address its role.

## Data Availability

The data that support the findings of this study are available from the corresponding authors upon request.

## Supplementary Materials

The following are available online at https://www.mdpi.com/article/10.3390/biom12010107/s1, Figure S1: TRPML2 silencing in RPMI cell line.
