# Supplementary material for: The Mucolipin TRPML2 Channel Enhances the Sensitivity of Multiple Myeloma Cell Lines to Ibrutinib and/or Bortezomib Treatment
Source: Biomolecules. 2022 Jan 9;12(1):107. doi: 10.3390/biom12010107 (PMC8773734; doi:10.3390/biom12010107)
Supplement: Supplementary file 1 [file biomolecules-12-00107-s001.zip › biomolecules-1535832-supplementary.pdf]

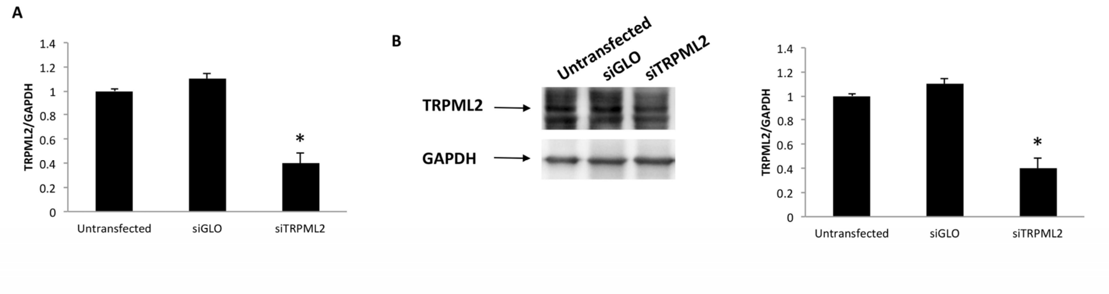

Supplementary Figure S1. TRPML2 silencing in RPMI cell line. A) The relative TRPML2 mRNA expression in untrasnfected, siGLO and siTRPML2 RPMI cells was evaluated by qRT-PCR. TRPML2 mRNA levels were normalized for GAPDH expression. Data are expressed as mean ± SD. *p<0.05 vs untransfectd and siGLO cells. B) Total lysates were separated on 8% SDS-PAGE and probed with anti-TRPML2 and anti-GAPDH Abs. TRPML-2 densitometry values were normalized to GAPDH used as loading control. Blots are representative of one of three separate experiments. *p < 0.01 vs untransfectd and siGLO cells.
